# Practical Observations on the Pathology and Treatment of Certain Diseases of the Skin, Generally Pronounced Incurable. Illustrated by Upwards of Forty Cases

**Published:** 1847-07

**Authors:** 


Art. II.—*Practical Observations on the Pathology and Treatment of certain Diseases of the Skin, generally pronounced Incurable. Illustrated by upwards of Forty Cases.* By Thomas Hunt, m.r.c.s., &c. —*London,* 1847. 8vo, pp. 156.

Mr. Hunt writes too much like one who indulges in the luxury of a hobby-horse, to gain assent to the whole of his views as to the power of arsenic in cutaneous diseases. He writes, however, in an earnest, truthful manner; and, making all allowance for the strong bias he exhibits towards his favorite remedy, the facts and views he brings forward eminently merit the attention of the practitioner.

The preparation of arsenic used by Mr. Hunt, is that commonly termed liquor arsenicalis, or Fowler's solution, the liquor potassæ arsenitis of the Pharmacopœia. With this remedy he has cured several forms of inveterate cutaneous disease; prurigo of the anus, scrotum, and pudendum, lepra, psoriasis, urticaria, impetigo, eczema, acne, sycosis, lupus exedens and nœvus araneus, are those in which he has found it successful, and some most obstinate cases are detailed as yielding to its powers.

The value of Mr. Hunt's publication consists rather, however, in the experience he has accumulated and given in it, as to the real effect of arsenic on the system, and as to the modes in which it should be administered. He advises, for example, that full doses should be administered at the first onset, as five minims of the solution thrice a day, and then that the doses should be diminished. The time for lowering the dose is indicated by the supervention of conjunctivitis: when a pricking sensation is experienced in the tarsi, the eyes suffused with tears, and the conjunctiva inflamed, the system is under the influence of the remedy, just as ptyalism indicates the full action of mercury. The skin on the trunk of the patient is also affected with slight inflammatory action, and subsequently the extremities, on a continued use of the medicine. This appears as a faint pityriasis, and all those parts of the surface protected from the light have a brownish, dingy, unwashed appearance. If this state be kept up, the disease will vanish just as rapidly as if the conjunctiva were kept sore.

Another observation (and one we think applicable to many other remedies besides arsenic) is, that the remedy is much less likely to affect the stomach or bowels if taken during a meal. With this we fully concur. The tolerance of the remedy varies much in different individuals. One patient was cured of a psoriasis guttata, *by a fourth part of a minim* of the solution taken thrice a day, or only $\frac{1}{480}$ of a grain of white oxide of arsenic.

We had noted some of the incidental results of the arsenical course to which Mr. Hunt submitted his patients, some of which were continued for two years. In several it appears to have acted as a vigorous tonic. We must, however, recommend the practitioner to read the book for himself; it will repay the trifling cost of time.

